# All oxide semiconductor-based bidirectional vertical p-n-p selectors for 3D stackable crossbar-array electronics

**DOI:** 10.1038/srep13362

**Published:** 2015-08-20

**Authors:** Yoon Cheol Bae, Ah Rahm Lee, Gwang Ho Baek, Je Bock Chung, Tae Yoon Kim, Jea Gun Park, Jin Pyo Hong

**Affiliations:** 1 Department of Nanoscale Semiconductor Engineering, Hanyang University, Seoul 133-791, Korea; 2 Department of Physics, Hanyang University, Seoul, 133-791, Korea; 3 Department of Electrical and Computer Engineering, Hanyang University, Seoul 133-791, Korea

## Abstract

Three-dimensional (3D) stackable memory devices including nano-scaled crossbar array are central for the realization of high-density non-volatile memory electronics. However, an essential sneak path issue affecting device performance in crossbar array remains a bottleneck and a grand challenge. Therefore, a suitable bidirectional selector as a two-way switch is required to facilitate a major breakthrough in the 3D crossbar array memory devices. Here, we show the excellent selectivity of all oxide p-/n-type semiconductor-based p-n-p open-based bipolar junction transistors as selectors in crossbar memory array. We report that bidirectional nonlinear characteristics of oxide p-n-p junctions can be highly enhanced by manipulating p-/n-type oxide semiconductor characteristics. We also propose an associated Zener tunneling mechanism that explains the unique features of our p-n-p selector. Our experimental findings are further extended to confirm the profound functionality of oxide p-n-p selectors integrated with several bipolar resistive switching memory elements working as storage nodes.

As conventional charge-based memories such as dynamic random access memory (DRAM) and NAND flash memory are rapidly approaching physical limitations, development of non-charge mechanism-based non-volatile memories (NVMs) is of great interest for numerous electronic applications^1^,^2^,^3^,^4^. Among the various NVMs considered recently, a resistive switching random access memory (ReRAM) is a highly reliable candidate to meet the demand of memory markets due to its high-density integration, long-retention time, small size, and fast-switching speed^4^,^5^,^6^,^7^,^8^,^9^. In particular, a simple metal-insulator-metal (MIM) frame employed in the ReRAM is expected to facilitate their use in nano-scalable crossbar arrays with ideal memory cell size (4F^2^) and multilayer stacking frames suitable for three-dimensional (3D) cells^10^,^11^,^12^,^13^. The crossbar array is typically composed of alternating bit-lines and word-lines perpendicular to each other with memory elements lying between them. However, as all cells in a row and column are connected to each other by the bottom and top electrode, the selected cell suffers from unintended leakage current arising from parasitic paths around neighboring cells. Therefore, the integration of a selector, such as a diode or transistor has been the focus of immense interest at each node, as illustrated in Fig. 1a^12^,^14^,^15^. In a commercial high-density memory system such as DRAM, a Si-based transistor has been used as a selector. However, the widespread use of three-terminal Si transistors is limited by the need for high processing temperature and difficulty in both scaling and stacking^16^,^17^. Therefore much effort has been dedicated towards the development of various suitable selectors, such as p-(n-)type Si-based n-p-n latch-up biristor devices, oxide tunneling based varistor-type devices, mixed-ionic-electronic-conduction devices, complementary resistive switching devices, MIM Schottky diodes, as well as metal-insulator transition and threshold switching devices^18^,^19^,^20^,^21^,^22^,^23^,^24^. However, several key issues still remain in the improvement of output performance, such as current density, cycling endurance, and distribution. Therefore, this work focuses on all oxide semiconductor-based p-n-p junctions as a generic approach for highly distinct bidirectional switches, possibly enabling their use in 3D scalable crossbar arrays including in conventional complementary metal-oxide-semiconductor processes. In particular, p-/n-type oxide semiconductors are one of the most likely sources that can be used at low processing temperatures, and they allow easy control of lattice mismatch and dopant impurities in p-n hetero-junctions which are the strong plausibility that such a device based on oxide semiconductors might be easy for the formation of the 3D stacable structures with good uniformity performance due to significant controllability margin in thickenss and doping profiles during growth.

We address the highly distinct electrical features of all oxide semiconductor-based p-CoO_x_/n-InGaZnO_x_/p-CoO_x_ open-based bipolar junction transistors for the bidirectional selectors, ensuring outstanding non-linear I-V behavior and promising stability. Systematic electrical analyses of the p-n-p junction selector were conducted by controlling the semiconductor characteristics of each p-(n-) type oxide semiconductor. We further propose a possible explanation of the unique outputs observed in the p-n-p selector based on a Zener tunneling event. Furthermore, we demonstrate the successful operation of a completely series-connected p-n-p selector for the one selector (1S) and bipolar resistive switching memory for the one resistor (1R) acting as a two-terminal 1S1R architecture, contributing to a general framework for advancing 3D crossbar array memory devices.

A detailed discussion of the sample preparation is given in the Methods section. All devices were sputter-deposited on bottom Pt/Ti electrodes on a commercially available SiO_2_/Si substrate. At first, a CoO_x_ layer serving as p-type oxide semiconductor was sputter-prepared by utilizing a CoO ceramic target in an oxygen and argon gas mixture. Then, an InGaZnO_x_ (IGZO) layer serving as an n-type oxide semiconductor was grown on the above CoO_x_ layer using a sputtering technique. Similarly, a top CoO_x_ layer was also deposited on the IGZO layer under the same growth conditions used for the bottom CoO_x_ layer. Finally, a top Pt electrode was defined using a conventional photolithography and lift-off process for 50 μm × 50 μm cell sizes.

## Results

### Resistive switching memory and p-n-p selector

**Figure 1:**
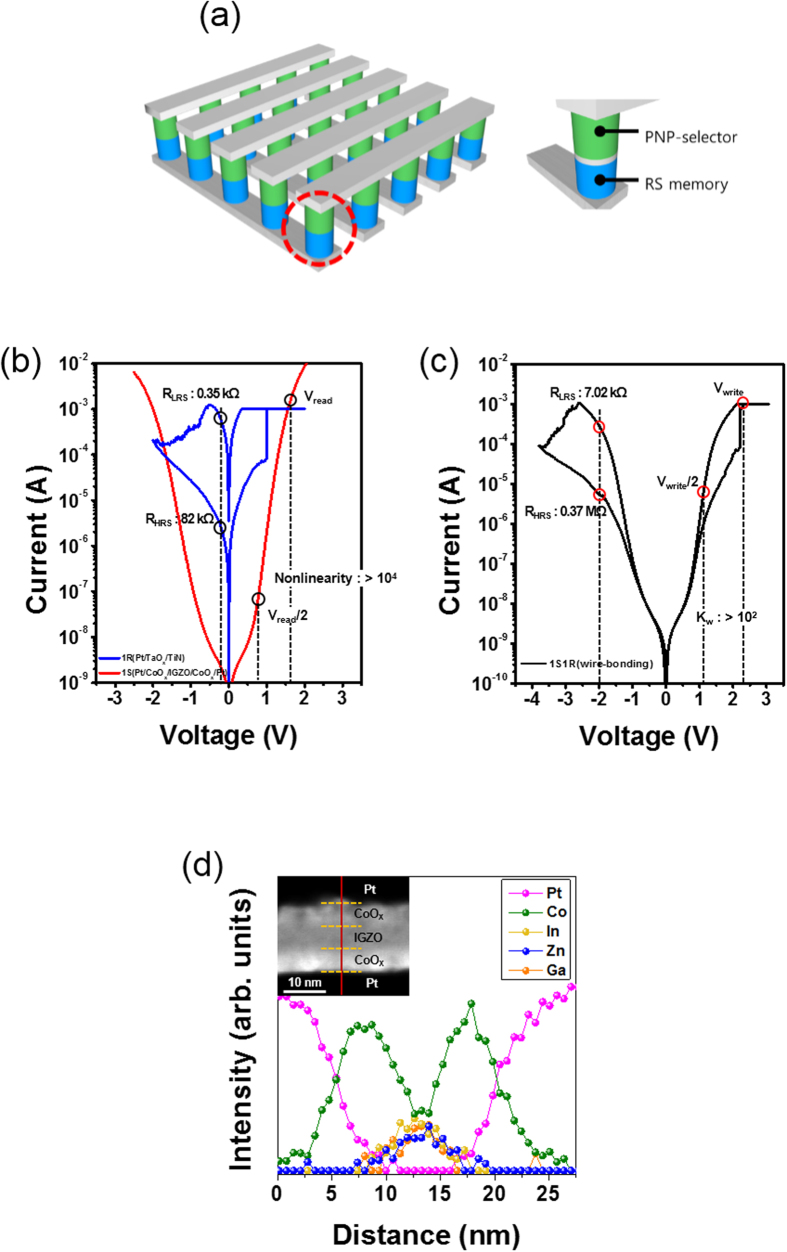
TiN/TaO_x_ resistive switching memory and CoO_x_/IGZO/CoO_x_ selector device. (**a**) Schematic of ideal crossbar array frame using memory and selector devices along with an enlarged view of unit cell. (**b**) Representative I-V characteristic of the 1S and 1R elements demonstrating the outstanding nonlinearity of over 10^4^ between V_read_ and V_read_/2 (V_read_ is at 1.6 V) and large memory window of over 10^2^. (**c**) Typical I-V characteristics of a serially connected selector and memory after completion of electrical connection, where a half voltage method was used. (**d**) High-resolution EDS (HR-EDS) elemental line profile across a line of the STEM image, where inset indicates a dark-field STEM image of the Pt/CoO_x_/IGZO/CoO_x_/Pt frame.

Figure 1a shows the ideal schematic crossbar array frame suitable for the most reliable stacking resistive memory elements with proper selectors between the crossbar metal bit and word-line electrodes. Individual I-V responses of typical p-CoO_x_/n-IGZO/p-CoO_x_ double-hetero-junction selectors (1S) and Pt/TaO_x_/TiN bipolar resistive switching elements (1R) are shown in Fig. 1b. As seen in this figure, the nonlinearity of the 1S selector is >10^4^ at a half-biased read scheme (V_read_ = 1.6 V and V_read_/2 = 0.8 V). The 1R memory element shows typical bipolar resistive switching behavior representing a larger memory window of 10^2^ at V_read_ = −0.2 V between a high resistance state (HRS) and low resistance state (LRS). Figure 1c shows the representative I-V curves of the serially connected 1S1R frame, where one electrode of the 1S selector is connected to the bottom TiN electrode of the 1R memory element. A sweeping bias was applied to the top Pt electrode of the 1S selector and was grounded to the top Pt electrode of the 1R memory element. During 1S1R operation, the set and reset voltages of the 1R memory element increased from 1.0 V to 2.3 V and −1.8 V to −3.7 V, respectively, due to the voltage drop across the 1S1R frame. In particular, the write nonlinearity (K_w_) of the 1S1R frame is >10^2^ at a half-biased write scheme of V_write_ = 2.3 V and V_write_/2 = 1.15 V, and the memory window of the 1S1R frame is >50 at V_read_ = −2 V. To determine the distribution of atomic profiles in the 1S selector, a cross-section scanning transmission electron microscopy (STEM) image and high resolution energy dispersive spectroscopy (HR-EDS) elemental line profile were obtained, and the results are shown in Fig. 1d. The HR-EDS elemental line profile illustrates clear Co atom distribution at the top and bottom CoO_x_ layers, along with In, Zn, and Ga atoms localized at the center of the IGZO layer. Further, a graded atomic composition at the interfaces between the CoO_x_ and IGZO layers of the p-n hetero-junction appears that is attributed to a gradually decreasing Co atom concentration and increasing In, Zn, and Ga atoms in the p- and n-oxide regions, respectively. In addition, a cross-sectional HR-EDS elemental mapping identifies noticeable well-grown triple multilayers of a 1S selector, as seen in Supplementary Figure S1. The inset of Fig. 1d provides a dark field STEM image of the 1S selector, confirming the formation of triple oxide layers.

### Dependence of p-n-p selector on propertises of n- and p-type oxide semiconductors

**Figure 2:**
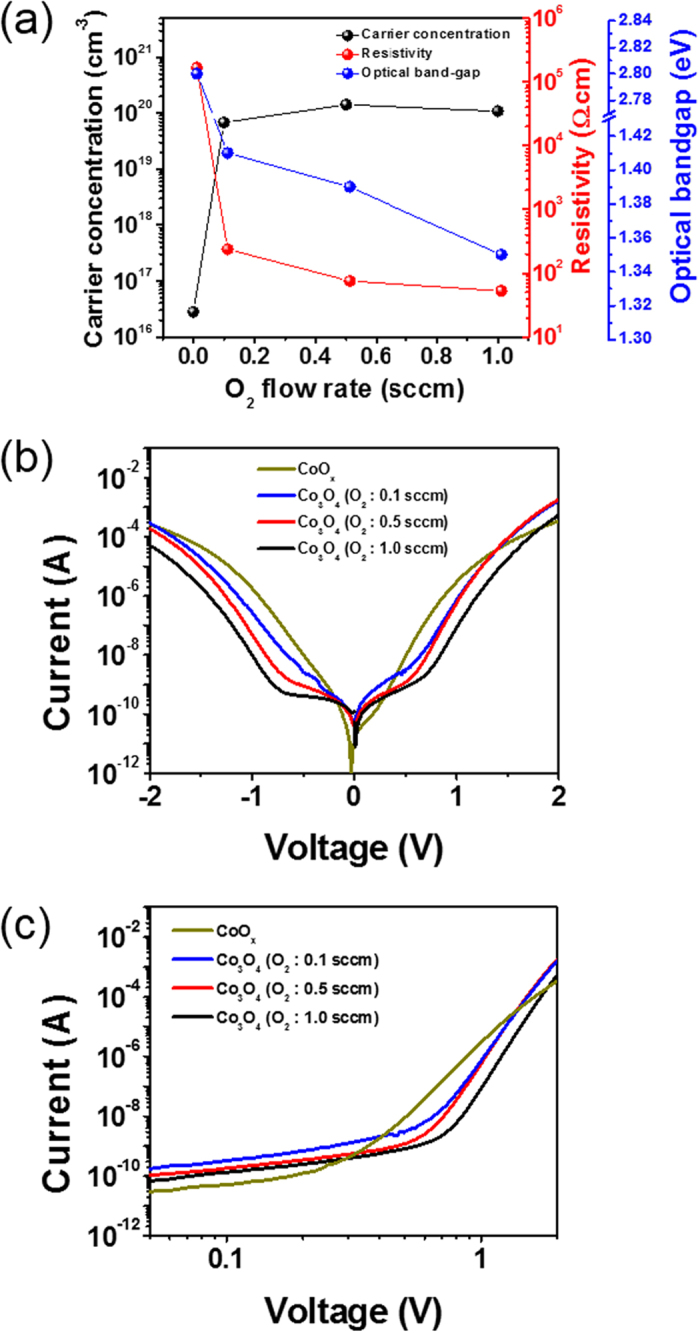
Effects of different p-type oxide semiconductor characteristics on the performance of a CoO_x_/IGZO/CoO_x_ selector. (**a**) Hall carrier concentration, resistivity and optical band gap of CoO_x_ thin films as a function of O_2_ flow rate. (**b**) I-V responses of several p-n-p selectors consisting of various CoO_x_ layers at a fixed IGZO layer. (**c**) Double-logarithmic scale plot taken at positive bias.

**Figure 3:**
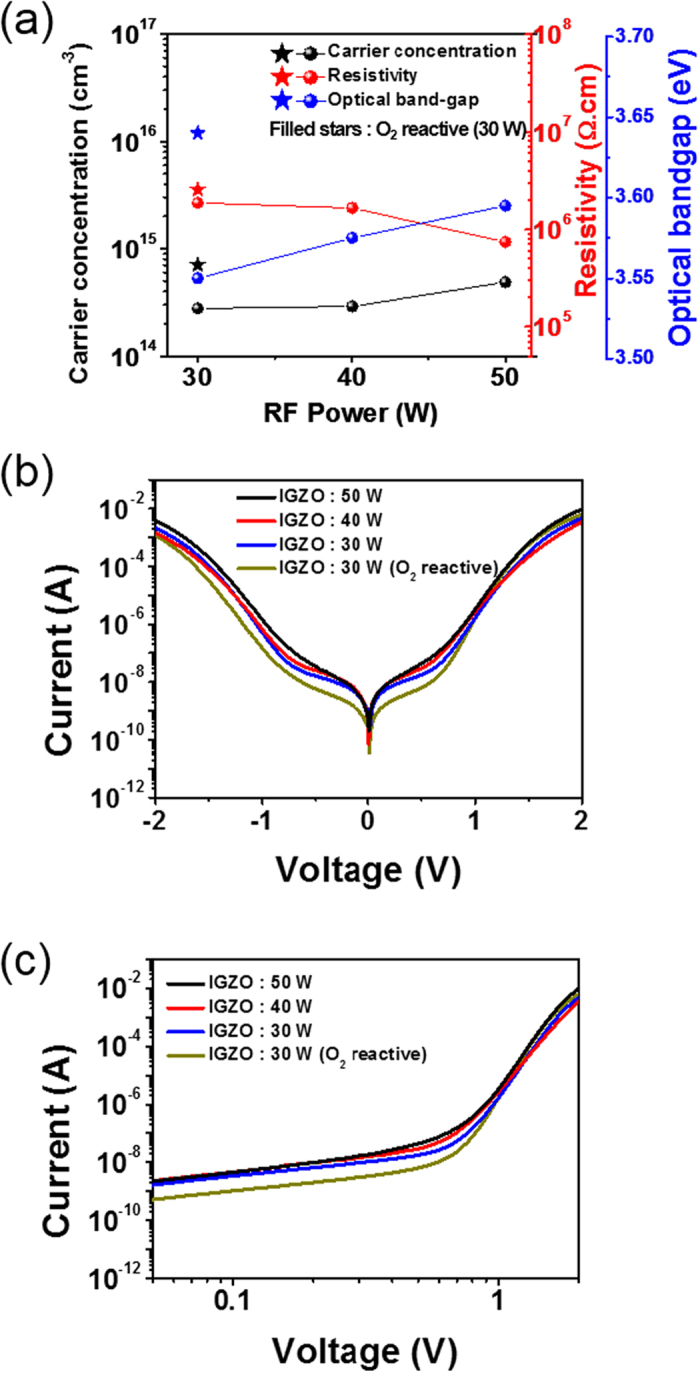
I-V characteristics of a CoO_x_/IGZO/CoO_x_ selector according to various n-type oxide semiconductor characteristics. (**a**) Hall carrier concentration, resistivity and optical band gap of IGZO thin films as a function of RF power. The physical features of representative O_2_ (0.2 sccm) reactive IGZO layer are indicated by filled stars. (**b**) I-V responses of several p-n-p selectors consisting of various IGZO layers at a fixed CoO_x_ layer. (**c**) Double-logarithmic scale plot taken at positive bias.

To clarify oxide semiconductor-dependent electrical features of a 1S selector, basic features of p-/n-type oxide semiconductors were examined as a function of oxygen content, RF power, and film thickness. Physical properties of p-/n-type oxide semiconductors were investigated by using Hall-Effect measurements (Figs 2a and 3a), X-ray diffraction (XRD) analysis (Supplementary Figure S2), and optical transmittance (Supplementary Figure S3). Furthermore, I-V features of each p-/n-type oxide semiconductor and oxide p-n junction were also analyzed in Supplementary Figure S4. Figure 2b,c reveal the resulting I-V features of 1S selectors containing different CoO_x_ layers, where the CoO_x_ layers are deposited under Ar and O_2_ (at various flow rates) with a fixed IGZO layer. For convenience, the CoO_x_ layer prepared under pure Ar is hereafter designated pure CoO_x_ and the CoO_x_ layers prepared under Ar/O_2_ flow are referred to as O_2_-reactive CoO_x_ layers. At first, the 1S selector (dark yellow color) with a pure CoO_x_ layer led to an exponential current increase over seven orders of magnitude for a voltage swing from 0 to ±2 V, providing a low off current (<100 pA) and a low turn-on voltage (~0.3 V). This low turn-on voltage results in a higher current level in the middle voltage region (~1.5 V) and earlier current saturation in the high voltage region (~2 V). This earlier current saturation induces a deteriorated nonlinearity in the 1S selector. The presence of low turn-on voltage and off-current can be described though an insulating characteristic present in a typical MIM selector that will be explained later^22^. However, the 1S selector containing an O_2_-reactive CoO_x_ layer reveals a highly nonlinear output with a remarkable difference in the current levels before and after application of a turn-on voltage. One striking feature is that all other selectors containing various O_2_-reactive CoO_x_ layers show promise in terms of the nonlinearity of the 1S selector. The off-state resistance, turn-on voltage, and slope of the double-logarithmic plot after turn-on voltage increase monotonically with increasing O_2_ flow rates. It is noteworthy that the I-V shape and its nonlinearity are highly affected by the introduction of oxygen gas during CoO_x_ deposition. Therefore, the XRD analysis for the CoO_x_ layer was carried out as a function of Ar and O_2_ flow rates, as given in Supplementary Figure S2a. In our work, CoO and Co_3_O_4_ phases are created in the only Ar and Ar/O_2_ mixture atmospheres, respectively. As is well-known from previous results reported by other groups, cobalt oxide typically has two stable phases: CoO and Co_3_O_4_^25^,^26^,^27^. The CoO phase represents a wide optical band gaps of 2.2–2.8 eV with an insulating feature, while the Co_3_O_4_ phase corresponds to narrow optical inter-band gaps of 1.4–1.5 eV and 2.18–2.23 eV with a semiconducting feature. Figure 2a shows the carrier concentration, resistivity, and optical band gap of CoO_x_ layers prepared at various O_2_ gas flow rates. Detailed optical band gap results of CoO_x_ layers are given in Supplementary Figure S3. Based on the above XRD analyses, our CoO phase shows insulating properties with a high resistivity of 165 kΩ·cm, a low carrier concentration of 2.8 × 10^16^ cm^−3^, and a wide band gap of 2.8 eV, while the Co_3_O_4_ phase shows semiconducting behavior with a low resistivity of 52–237 Ω·cm, a high carrier concentration of 6.8 × 10^16^–1.4 × 10^21^ cm^−3^, and a narrow inter band gap of 1.3–1.4 eV. The above findings are consistent with the references for CoO and Co_3_O_4_^25^,^26^,^27^,^28^,^29^. In addition, increasing O_2_ flow rate reflects enhanced semiconductor characteristics for the CoO_x_ layer including an increased carrier concentration and a decreased optical band gap. Thus, the 1S selector containing a pure CoO_x_ layer is mainly governed by the insulating CoO monoxide phase working as an insulator, not a p-type oxide semiconductor. In contrast, the nonlinear characteristic of the 1S selectors containing an O_2_-reactive CoO_x_ layer is mainly attributed to the semiconducting Co_3_O_4_ sub-oxide phase. It is widely believed that a large carrier concentration in a collector determines the presence of Zener breakdown between the base and collector in a bipolar junction transistor, and a small band gap in the p-side of an oxide p-n junction diode causes increased band offset in the reverse bias region^30^,^31^,^32^,^33^. Thus, the introduction of oxygen during growth in our work positively governs the CoO_x_ semiconducting feature, demonstrating the enhanced nonlinearity of the 1S selector by promoting lower off-current, increased turn-on voltage, and exponentially increased turn-on current.

To further access the electrical characteristics of 1S selectors according to various IGZO layers, similar measurements are made by varying RF power and oxygen partial pressure during IGZO growth. All four IGZO layers reveal amorphous phases, as shown in Supplementary Figure S2b. Figure 3a provides the optical band gap and carrier concentration of the IGZO layer as a function of RF power. Decreasing RF power results in decreased optical band gap, electrical conductivity, and carrier concentration. These features are well known in the oxide thin film transistor industry^34^. However, the introduction of oxygen (0.2 sccm O_2_ flow rate) during IGZO growth increases carrier concentration, resistivity, and optical band gap. In general, the resistivity of the n-IGZO layer relies on both mobility and carrier concentration. In our work, the O_2_-reacitve IGZO layer provides a slightly increased carrier concentration (3 × 10^14^ → 6 × 10^14^ cm^−3^) and a sharp decrease in mobility (0.012 → 0.0034 cm^2^/V·s, see in Supplementary Figure S5). Thus, the achievement of increased resistivity in an O_2_-reactive IGZO layer is likely due to a low mobility, resulting in the increased resistivity of a 1S frame suitable for the creation of a low off-current. Figure 3b,c show representative I-V responses of 1S selectors including various IGZO layers. The off-state, on-current, and I-V slope after turn-on voltage slightly decrease with decreasing RF power, but the turn-on voltages remain almost unchanged because of a compensation effect between the decreased band gap and carrier concentration on the n-side with increased resistivity. However, when an O_2_-reactive IGZO layer with an increased carrier concentration, band gap, and resistivity is involved in a 1S selector, the 1S selector shows a low off-current and a higher I-V slope after application of a turn-on voltage. We expect that the increase in a carrier concentration of the IGZO layer significantly contributes to the reduction of both the depletion region inside the IGZO and the triangular barrier width in a reversed p-n junction. In addition, an increase in the band gap of IGZO reflects enhanced band offset in the p-n junction. Therefore, both increased height and decreased triangular barrier width in a reversed p-n junction suggest a Zener tunneling event that is directly linked to the enhanced nonlinearity of the 1S selector containing an O_2_-reactive IGZO layer, as shown in Fig. 3b.

### Oxide thickness dependence and switching mechanism

**Figure 4:**
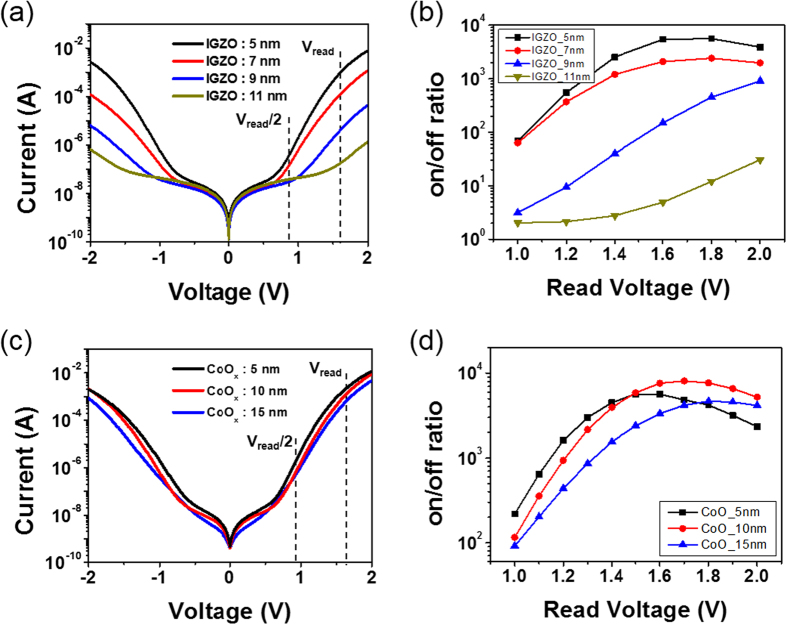
Oxide semiconductor thickness dependence of a CoO_x_/IGZO/CoO_x_ selector. (**a**) I-V responses of p-n-p selectors containing 5-, 7-, 9-, and 11-nm-thick IGZO layer with a 5-nm-thick CoO_x_ p-type oxide semiconductor. (**b**) On/off ratios of each selector recorded at V_read_ and V_read_/2. (**c**) I-V features of 5, 10, 15 thick CoO_x_ with a 5-nm-thick IGZO n-type oxide semiconductor. (**d**) On/off ratio of each selector taken at V_read_ and V_read_/2.

To gain insight into the role of p- or n-type oxide semiconductor thickness on the output of the 1S selector, various n-/p-type oxide thicknesses were also examined, as shown in Fig. 4. The 1S selector is composed of an oxygen reactive (1.0 sccm) CoO_x_ layer and a non-oxygen reactive IGZO layer. Figure 4a show the I-V responses of 1S selectors containing various IGZO thicknesses, suggesting the strong dependence of turn-on voltage and on-currents in the IGZO thickness. However, the 1S selectors with various CoO_x_ thicknesses are not significantly affected, as shown in Fig. 4c. The on/off ratios of 1S selectors with different IGZO and CoO_x_ thicknesses are determined at different read voltages in a half-biased read scheme, as demonstrated in Fig. 4b,d. A decrease in IGZO thickness leads to improved nonlinearity in the 1S selector by allowing a decrease in a maximum read voltage (~1.6 V), as shown in Fig. 4b. However, as seen in Fig. 4d, the highest nonlinearity is achieved for a 10-nm-thick CoO_x_ layer because the nonlinearity of the 1S selector containing a relatively thin CoO_x_ of about 5 nm is saturated at a low read voltage (~1.4 V) and decreases rapidly thereafter. In general, the conductivity in a 1S selector relies on the total resistance of device after turn-on. In our work, a thick CoO_x_ layer provides an increased resistance of 1S, reflecting a slight decrease in on-current without affecting turn-on voltage. In addition, because a higher carrier concentration in a CoO_x_ layer (rather than an IGZO layer) corresponds to a depletion region inside the IGZO layer, most energy band bending and Zener tunneling may occur in the depletion region of the IGZO layer. Consequently, the thickness of the n-type IGZO layer is likely responsible for the resulting electrical key factors, such as turn-on voltage and on current, as confirmed in Fig. 4a.

**Figure 5:**
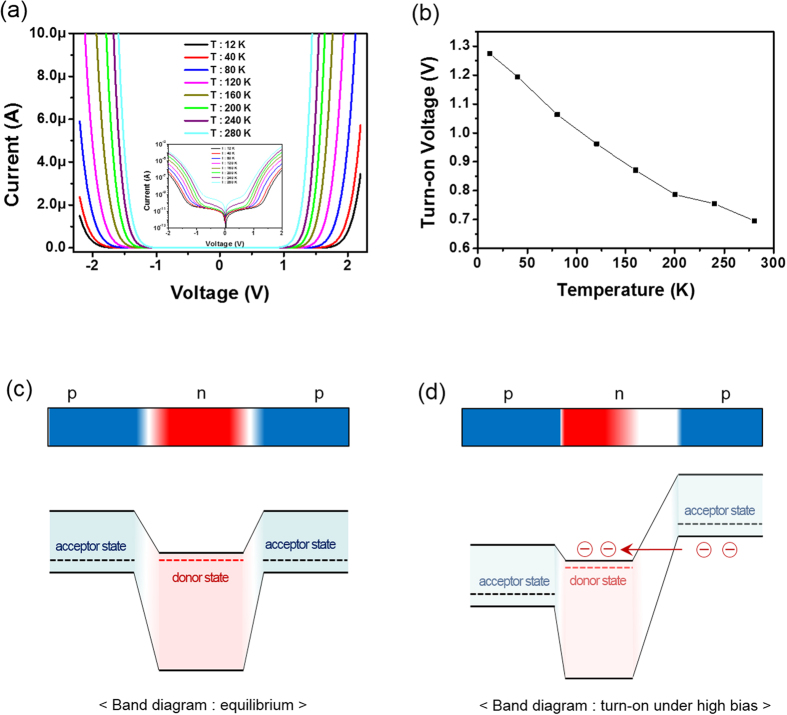
Temperature-dependent I-V features and schematic diagram for a CoO_x_/IGZO/CoO_x_ selector. The p-n junctions were described, based on graded composition of hetero-junctions: (**a**) I-V features recorded from 12 K to 280 K and (**b**) temperature dependence of turn-on voltage. (**c**) Schematic diagram in equilibrium and (**d**) high bias conditions including depletion region width, band diagram, and electron flow. The insets in (**a**) show semi-log temperature dependence I-V curve of CoO_x_/IGZO/CoO_x_ selector.

To further identify whether the proposed Zener tunneling is responsible for electrical observations in this work, temperature measurements on our p-n-p selectors were also conducted and the results is shown in Fig. 5a,b. It is well-known that typical junction breakdown events are caused by either Zener tunneling or avalanche multiplication, in which the Zener tunneling breakdown voltage has a negative temperature coefficient whereas the avalanche has a positive temperature coefficient. A gradual decrease in turn-on voltage of p-n-p selector with increasing temperatures was observed, as shown in Fig. 5b. It clearly reflects the occurrence of Zener tunneling event. The inset in Fig. 5a was the I-V plot of 1S frame in a semi-log scale. Based on the above observations and low temperature measurement, we propose a possible model to explain the electrical behavior of bidirectional 1S selectors by means of a reverse breakdown event, as illustrated in Fig. 5c,d. At first, we hypothesize that the bidirectional nonlinear behavior in the p-n-p selector is analogous to the Zener tunneling presented in an open-base n-p-n double hetero-junction bipolar transistors^30^,^31^. Figure 5c shows the equilibrium band diagram, suggesting suppressed current flow at zero and low bias voltage since the reversed bias p-n junction exists inherently in a p-n-p selector, regardless of the applied positive or negative bias to the top (or bottom) CoO_x_ layer. In our model for a p-n hetero-junction, the role of any potential well is neglected due to the formation of a graded composition at the IGZO/CoO_x_ interface, as shown in Fig. 1d. However, when a specific voltage is applied to the top (or bottom) CoO_x_ layer, the staggered band lines up between the p-type CoO_x_ valence band edge and n-type IGZO conduction band edge, giving rise to the presence of a narrow effective gap at the reversed p-n junction. Therefore, Zener tunneling is likely to occur at a narrow effective gap, as seen in Fig. 5d. Moreover, since the depletion region is primarily created towards the n-type IGZO side, the turn-on voltage is largely governed by the IGZO thickness. The above findings are correlated with the findings in Fig. 4. If an n-type IGZO layer is thinner than the depletion region, Zener tunneling can appear even at a relatively low bias voltage. Thus, the 1S selector acts as a constant resistor with a higher off-current and a low turn-on voltage. Additional thickness dependence measurements confirmed a linear I-V behavior of a 1S frame with a 2-nm-thick IGZO layer, as plotted in Supplementary Figure S6.

### Performance of 1S1R integration

**Figure 6:**
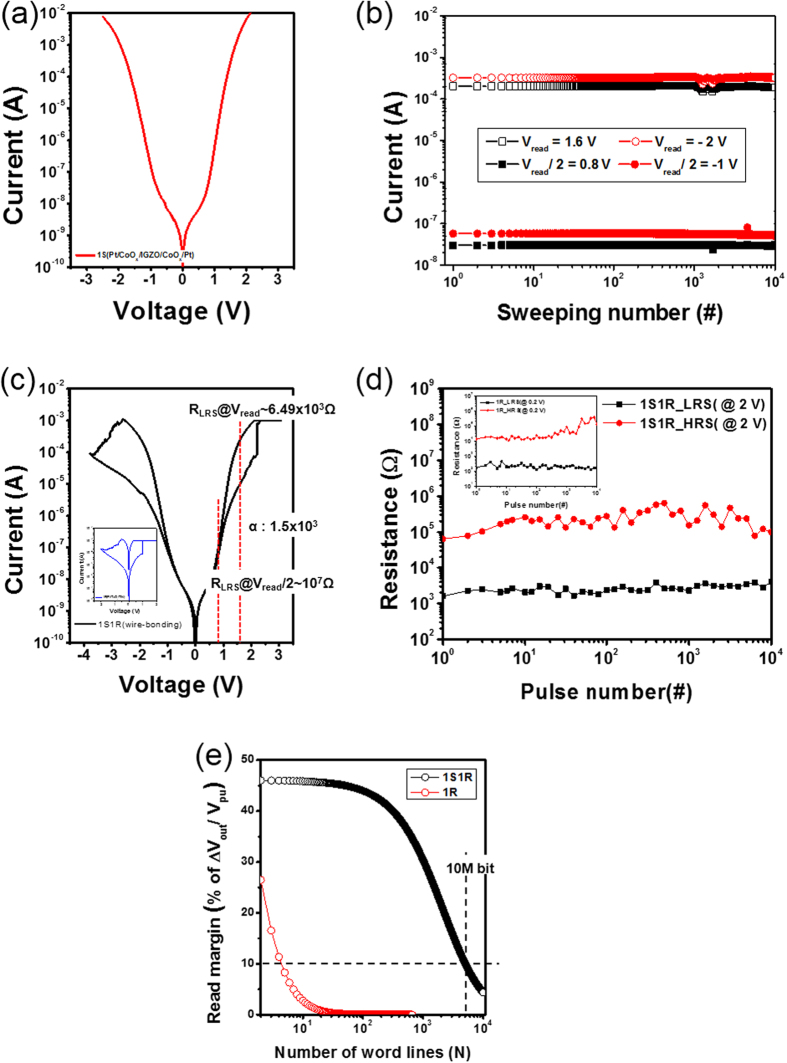
Characteristics of 1S1R frame. (**a**) Typical I-V characteristics of a CoO_x_/IGZO/CoO_x_ selector and (**b**) endurance test under 10^4^ consecutive DC sweeps. (**c**) Typical I-V characteristics of Pt/CoO_x_/IGZO/CoO_x_/Pt (1S) integrated with Pt/TaO_x_/TiN (1R) after completion of electrical connection. (**d**) Endurance test measured by a 100-ns pulse width signal. (**e**) Dependence of normalized read margin (ΔV_out_/V_pu_) on the crossbar line number (N) for 1R (red color) and 1S1R (Black color) devices. The insets of (**c**,**d**) show I-V curve and endurance characteristics of only 1R.’

To highlight the suitability of the 1S selector developed in this work, the electrical features of 1S1R frames integrated with various 1R memory elements are systematically examined, where the optimized 1S selector consists of O_2_-reactive 10-nm-thick p-CoO_x_ (1.0 sccm) and 5-nm-thick n-IGZO (0.2 sccm) layers. Figure 6a,b reveal typical I-V responses and a dc endurance test of only the 1S selector, where the endurance test is conducted at a half-bias read voltage scheme (V_read_ = 1.6 V and −2 V, V_read_/2 = 0.8 V and −1 V). The selectors exhibit promising stability at V_read_ and V_read_/2 during 10^4^ consecutive DC sweeps. Figure 6c,d show the representative I-V switching response and pulse endurance results of the 1S1R frame. In a dc bias sweep, the set and reset voltages of 1S1R frame are 2.4 and −3.8 V, respectively, as seen in Fig. 6c. A voltage pulse diagram for the endurance test is shown in Supplementary Figure S7, where the amplitudes of set and reset voltages are 3 and −5.6 V, respectively. The nonlinearity factor (α) in Fig. 6c is defined as the ratio of LRS resistances measured at V_read_ and V_read_/2, demonstrating a value of more than 10^3^, which is comparable to other nonlinear selectors^19^,^22^,^35^. To further estimate the possible array sizes for our 1S1R frames, a normalized read voltage margin (ΔV_out_/V_pu_) is calculated by using a number of word lines, as seen in Fig. 6e. The cell condition assumes the worst read scheme, called a one bit-line pull up scheme^36^,^37^,^38^. For only a 1R memory element, the measurable normalized read voltage margin ΔV_out_/V_pu_ rapidly decreases to 10% for the N = 5 array size (N-word line and N-bit line). However, the calculated maximum array size of a 10% read voltage margin is extended to N = 5067 for the 1S1R frame, meaning an array size of more than 10 Mbit is possible. In addition, the endurance tests of 1R and 1S1R frames demonstrate a negligible degradation in the memory window during successive operation of 10^4^ pulses. However, even though a highly uniform dc endurance feature is obtained for a 1S frame, a detectable fluctuation is observed in a pulse endurance test of a 1S1R frame. This is mainly due to the disturbed endurance fluctuations of 1R memory elements, as shown in the inset of Fig. 6d. Therefore, 1S1R device characteristics strongly rely on the electrical features of the 1R memory element, such as distribution of endurance, retention, and memory window since the 1S frame is highly stable. In addition, multi-level switching behavior arising from both 1R memory elements is similarly confirmed in 1S1R frames, as seen in Supplementary Figure S8. In particular, to validate the possible use of our 1S selector as a bidirectional switch, the I-V responses of 1S selectors integrated with various memory elements are observed, as seen in Supplementary Figure S9. All devices exhibit good nonlinearities, as expected. As given in Supplementary Figure S9d, the conducting bridge memory (CBM) elements provide a large 1S1R memory window of more than 10^3^, along with the relatively unstable features caused by larger fluctuations frequently present in the CBM 1R memory element. In addition, the 170 nm nano-scaled p-n-p selectors in this work were examined, resulting in the current density of 0.1 MA/cm^2^ (Supplementary Figure S10). Thus, we expect that there is still a lot of space to be improved after optimization process as a two-way switch for NVMs. Furthermore, we have developed the p-n-p oxide selector by using a semiconductor industry-friendly TiN electrode. However, since the TiN electrode with a reactive characteristic is highly susceptible to reaction with bottom or top CoO_x_ layers, resulting in the degraded electrical performance, a tin-doped indum oxide (ITO) layer was inserted between TiN and bottom CoO_x_ to block the possible reaction of oxide materials with reactive electrode (Supplementary Figure S12).

In summary, we described the outstanding performance of 1S selectors involving all oxide semiconductor materials as a promising alternative to solve sneak-path issues possibly present in 3D stackable nano-scaled crossbar memory array configurations. We examined the p-/n-type oxide semiconductor material-dependent nonlinearity of a 1S selector, along with a physical model to explain the nonlinear I-V features observed. In particular, the 1S1R frame integrated with various 1R memory elements confirms the possible suppression of the leakage current when compared to that of the 1R memory element, ensuring stable switching endurance up to 10^4^ cycles with a 100-ns pulse width. We anticipate that this approach will become a simple and useful route that offers the possible realization of future 3D stackable crossbar array memory devices.

## Methods

### Measurement

DC electrical measurement was performed by using a Keithley 4200 semiconductor parameter analyzer (Keithley 4200 SPA, Keithley Instruments, Inc.). Temperature dependent characteristics were recorded using a Helix CTI-Cryogenic 8200 compressor (Helix Technology Corporation) and a Neocera LTC-11 temperature controller under vacuum. Pulse analyses for cycling endurance were carried out with an Agilent 81110A pulse generator (Agilent Technologies, Inc.). Hall Effect measurement was conducted at room temperature by utilizing a standard set-up using the Van der Pauw method with a Lakeshore 662 electromagnet power supply and a Keithley 4200 SPA. The crystalline features of oxide layers were characterized by using an X-ray diffraction system (XRD, Rigaku D/MAX-2500/PC, by Cu Kα radiation). Optical absorption was recorded using a UV-visible spectrometer (Lambda 35: PerkinElmer). Cross-sectional nanostructured observation and atomic distribution analyses of a selector were conducted by using a HR-STEM (JEOL JEM 2100F) with the corresponding EDS mappings. For HR-STEM measurements, across cutting sample was prepared on a copper grid using a focused ion beam (FIB).

### Fabrication

Various Pt/TaO_x_/TiN, Ta/TaO_x_/Pt and Cu/TaO_x_/Pt bipolar resistive switching memory device and Pt/CoO_x_/IGZO/CoO_x_/Pt bidirectional selector device were fabricated on commercially available SiO_2_/Si substrates. A 30-nm-thick Ti layer under the Pt bottom electrode was prepared as a buffer layer on the SiO_2_ by using a sputtering system. The bottom TiN electrode was formed by reactive sputtering of a Ti metal target in a mixture of Ar and N_2_. All oxide layers were grown by using RF magnetron sputtering with ceramic targets. A 20-nm-thick TaO_x_ oxide layer was chosen as the main active medium suitable for memory elements and then various 50-nm-thick top electrodes (Pt, Ta, and Cu) were formed by sputtering of metal targets with a 50 μm × 50 μm cell size defined by photolithography and a lift-off process. Furthermore, a 170 nm-size p-n-p selector were prepared on the TiN nano-pluged wafer (Supplementary Figure S11).

## Additional Information

**How to cite this article**: Bae, Y. C. *et al*. All oxide semiconductor-based bidirectional vertical p-n-p selectors for 3D stackable crossbar-array electronics. *Sci. Rep*. **5**, 13362; doi: 10.1038/srep13362 (2015).

## Supplementary Material

Supplementary Information
