# Supplementary material for: All oxide semiconductor-based bidirectional vertical p-n-p selectors for 3D stackable crossbar-array electronics
Source: Sci Rep. 2015 Aug 20;5:13362. doi: 10.1038/srep13362 (PMC4542327; doi:10.1038/srep13362)
Supplement: Supplementary Information [file srep13362-s1.doc]

**Supplementary Information for “All oxide semiconductor-based bidirectional vertical p-n-p selectors for 3D stackable crossbar-array electronics”**

Yoon Cheol Bae^1^, Ah Rahm Lee^1^, Gwang Ho Baek^1^, Je Bock Chung^1^, Tae Yoon Kim^2^, Jea Gun Park^1,3^, and Jin Pyo Hong^1,2★^

*^1^Department of Nanoscale Semiconductor Engineering, Hanyang University, Seoul 133-791, Korea*

*^2^Department of Physics, Hanyang University, Seoul, 133-791, Korea*

*^3^Department of Electrical and Computer Engineering, Hanyang University, Seoul 133-791, Korea*

**Corresponding author E-mail:** [**jphong@hanyang.ac.kr**](mailto:jphong@hanyang.ac.kr)

Supplementary figures


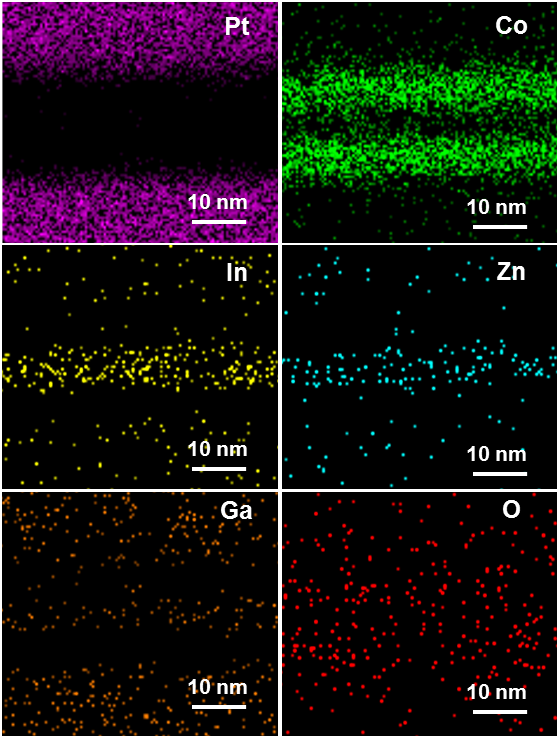
1. Elemental mapping analysis of Pt/CoO_x_/IGZO/CoO_x_/Pt

Figure S1. HR-EDS elemental mapping images for the Pt/CoO_x_/IGZO/CoO_x_/Pt frame, indicating spatial distribution of Pt (pink), Co (green), In (yellow), Zn (blue), Ga (orange), and O (red). The preparation was conducted by utilizing a focused ion beam system with a Ga source.

Figure S1 shows the HR-EDS elemental mapping of an STEM image region taken in Fig. 1d. The Co, In, Zn elemental mapping images clearly reveal the presence of three PNP oxide layers between the top and bottom Pt electrode, where the O element appears through the whole PNP oxide region. Ga atoms in the top and bottom Pt electrode regions were also observed, mainly arising from the sample preparation used for the STEM image by the focused ion beam process, not due to the inter-diffusion of other elements towards the Pt electrode.


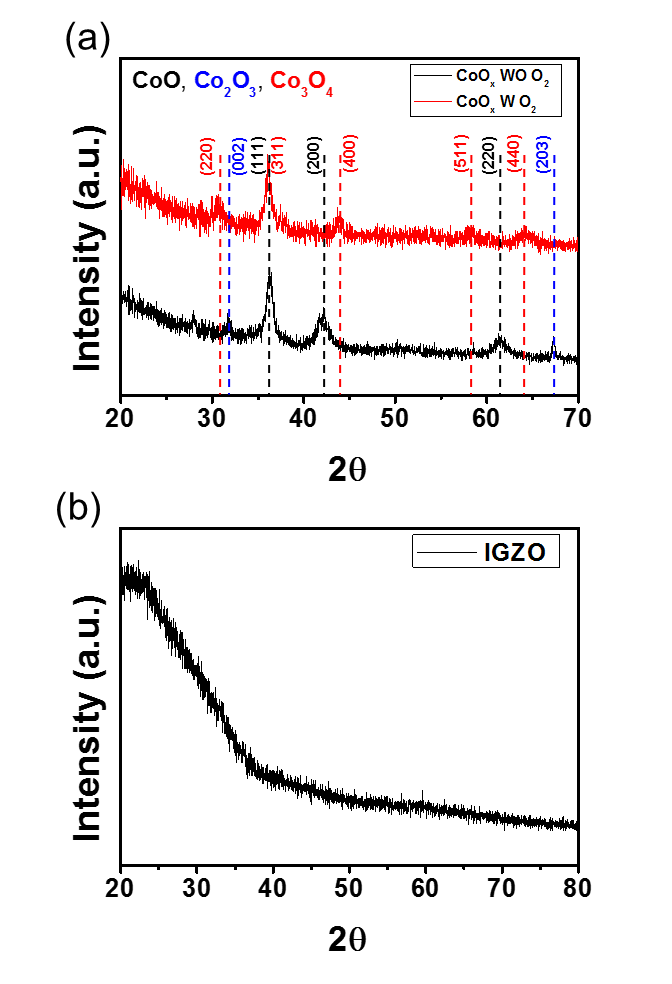
2. Structural characteristics of p- and n-type oxide semiconductors

Figure S2. XRD patterns of P-type CoO_x_ and N-type IGZO layers. (a) XRD patterns of CoO_x_ thin films deposited without/with O_2_ gas flow in only Ar (15 sccm) and Ar (15 sccm)/O_2_ (0.1 sccm) mixture atmospheres. (b) XRD pattern of IGZO thin film prepared in only Kr (15 sccm) gas atmosphere.


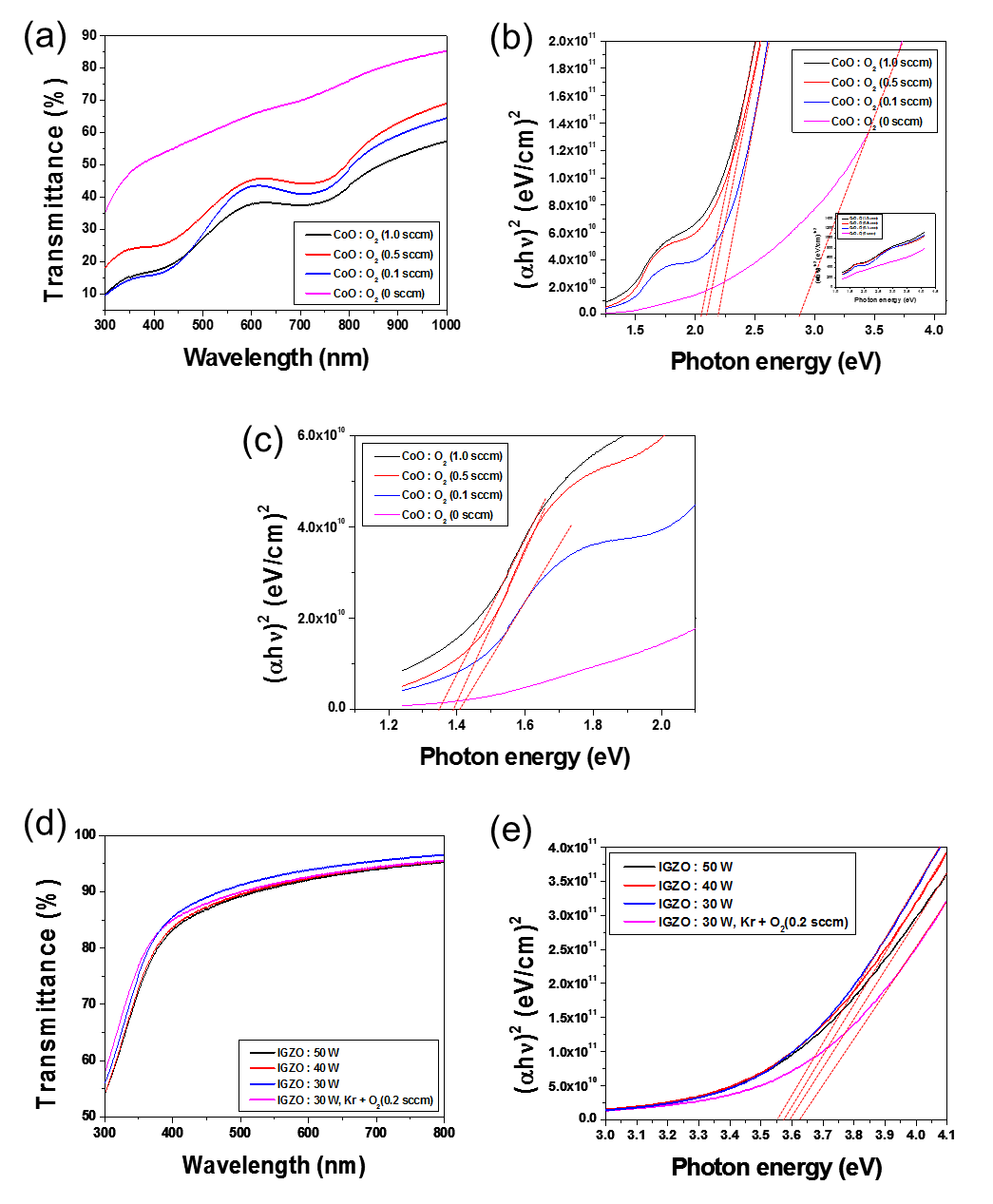
3. UV-transmittance spectra of oxide semiconductor

Figure S3. Optical transmittance spectra of p and n-type oxide semiconductor deposited on glass substrates. (a) Transmittance spectra of CoO_x_ prepared at various O_2_ flow rates. (b) and (c) Tauc plots of optical absorption coefficients for the CoO_x_ layers taken at different photon regions. (d) Transmittance spectra of IGZO layers grown at different RF powers and O_2_ flow rates. (e) Tauc plot of the optical absorption coefficients for the IGZO. The insets in (b) show plots of (αhʋ)^1/2^ = f(hʋ) for CoO_x_ in in-direct transition.

Figure S3 shows the optical transmittance spectra and absorption coefficient (α) power law of oxide semiconductors. All oxide semiconductors were deposited on glass substrates for the measurements. Figure S3a presents two absorption bands centered at 410 and 750 nm, absorption band centers of Co_3_O_4_ associated with a charge transfer transition of *p*(O^2-^) → *e_g_*(Co^3+^) and Co^3+^ → Co^2+^, respectively. To better understand the CoO_x_ samples, the power-law dependence of the optical transmission spectra is re-plotted, as indicated in Fig. S3b and c. Incident photon energy has a relationship of αhʋ = A(hʋ – E_g_)^n^, where α = − 1/d ln(1/T), d, T, and hʋ represent the absorption coefficient, thickness of CoO_x_, transmittance, and photon energy, respectively. E_g_ is the direct and indirect band gaps for n = 1/2 and n = 2, respectively. The E_g_ is determined by extrapolating the linear region to the photon energy axis. The O_2_ reactive CoO_x_ films represent two direct band gaps of E_g_ = 1.35-1.41 eV and 2.05-2.2 eV, as seen in Fig. S3b and c. Indirect band gaps are also extrapolated in the inset of Fig. S3b, exhibiting a decreased band gap. For the n-type oxide IGZO, the optical transitions are simpler, as seen in Fig. S3d and e. The IGZO layer reveals only a direct transition at E_g_ = 3.52-3.64 eV, and the band gaps increased with increasing RF power and O_2_ insertion during deposition. The increase in E_g_ can be well-described by the Burstein-Moss effect. That is, when the conduction band becomes significantly filled at a high doping concentration and the lowest energy states in the conduction band are blocked, the E_opt_ is enhanced in n-type semiconductors. In addition, the Hall Effect observations indicate a strong variation in the carrier concentration and optical band gap with increasing RF power and O_2_ insertion during deposition, as mentioned in Fig. 3a.


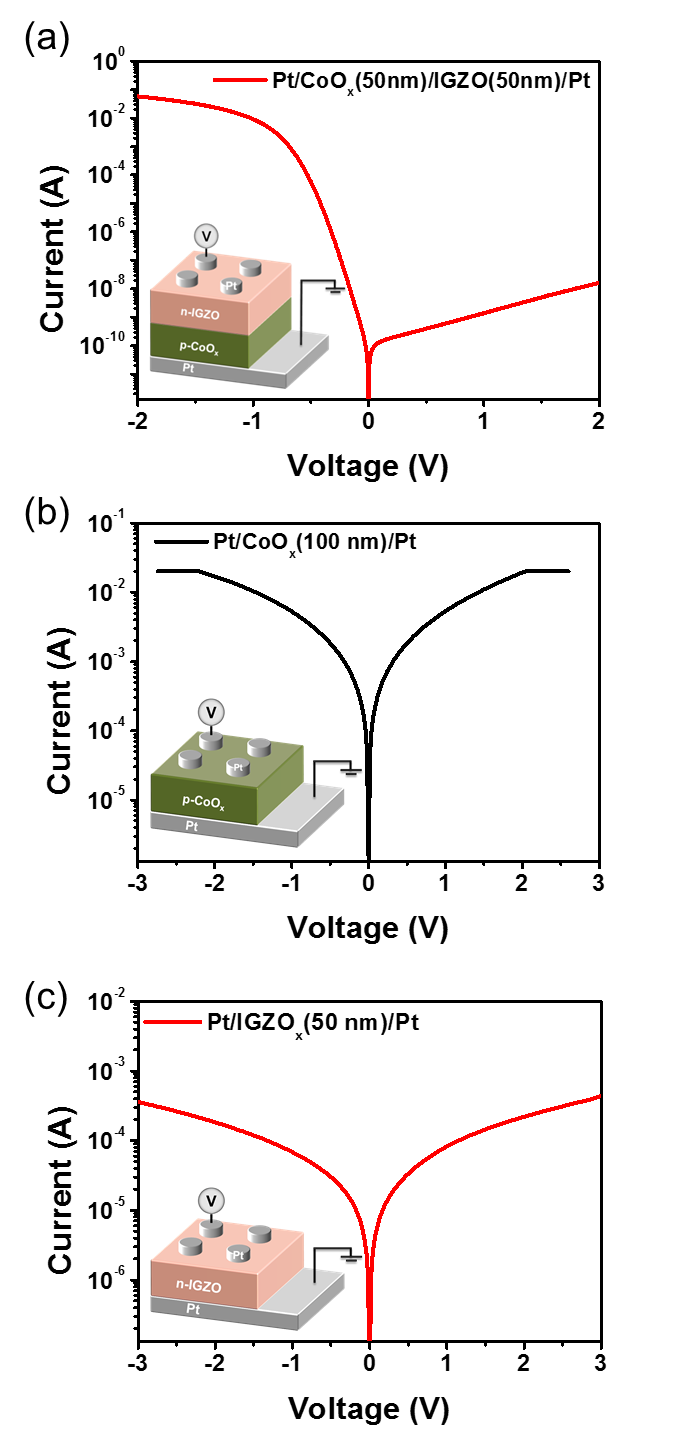
4. Electrical characteristics of p-n junction and each single oxide semiconductors

Figure S4. I-V features of single oxide p-n diode and capacitor frames. (a) I-V characteristic of single Pt/CoO_x_/IGZO/Pt p-n frame demonstrating the rectifying features, where a voltage was applied across the top Pt electrode and the bottom Pt electrode was grounded. (b) and (c) representative I-V response of Pt/CoO_x_/Pt and Pt/IGZO/Pt frame acting as a resistor, respectively. The insets in (a), (b), and (c) represent schematics of one p-n diode and two resistors, respectively.


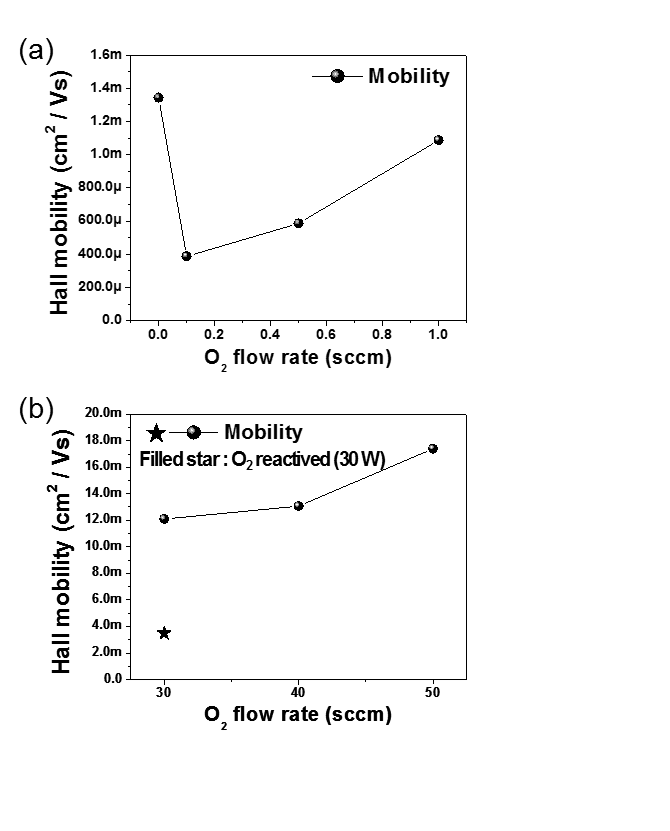
5. Hall mobility of each p- and n-type oxide semiconductor

Figure S5. Hall mobility of p, n-type oxide semiconductors deposited on SiO_2_/Si substrates for (a) CoO_x_ thin films as a function of O_2_ flow rate and (b) IGZO thin films as a function of RF power, in which the Hall mobility of O_2_ (0.2 sccm) reactive IGZO thin films was defined as a filled star.


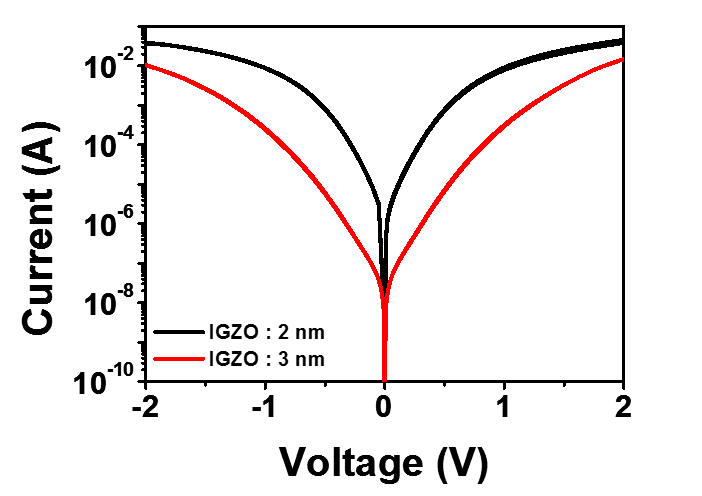
6. Electrical characteristics of thinner IGZO p-n-p selector

Figure S6. Ultrathin IGZO thickness contribution to the performance of 1S selector. I-V characteristics of 1S selector containing ultrathin 2- (black line) and 3-nm-thick (red line) IGZO layers at a fixed 5-nm-thick CoO_x_ p-type layer, demonstrating the resistive behavior. This resistive phenomenon is directly due to the presence of the ultrathin IGZO layer that is a comparable thickness or less than that of the possible depletion region occurring inside the IGZO layer.


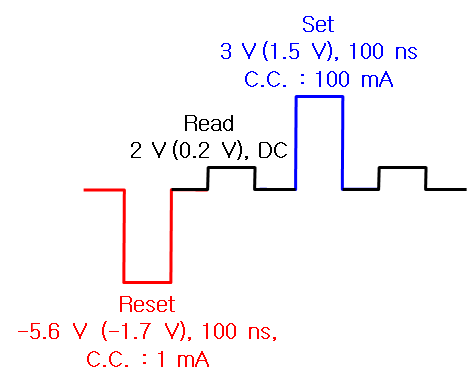
7. Pulse endurance test

Figure S7. Schematic illustration of the voltage pulse for endurance test. Set and reset process operated at pulse and DC voltages, respectively.

Figure S6 shows a schematic voltage pulse diagram used for the endurance test, where a 100-ns-pulse width was selected as a set and reset pulse using an Agilent 81110A pulse generator. The reset and set pulse amplitudes for 1R memory elements are − 1.7 and 1.5 V, respectively, which are higher than those determined in dc voltage operation. The measurement of the 1S1R frame involves pulse reset and set amplitudes that are increased up to − 5.6 and 3 V, respectively. In particular, the currents are limited up to 1 mA and 100 mA as compliance currents (C.C.) for set and reset processes. For cycling endurance measurements, the impedance of the pulse generator varied during the set and reset process. After each set and reset pulse was applied, the cell states were identified by using a dc read voltage with Keithley 4200 SPA and Keithley 708A switching systems used for switching pulse and dc voltages, respectively. The endurance test is repeated up to 10^4^ switching cycles.


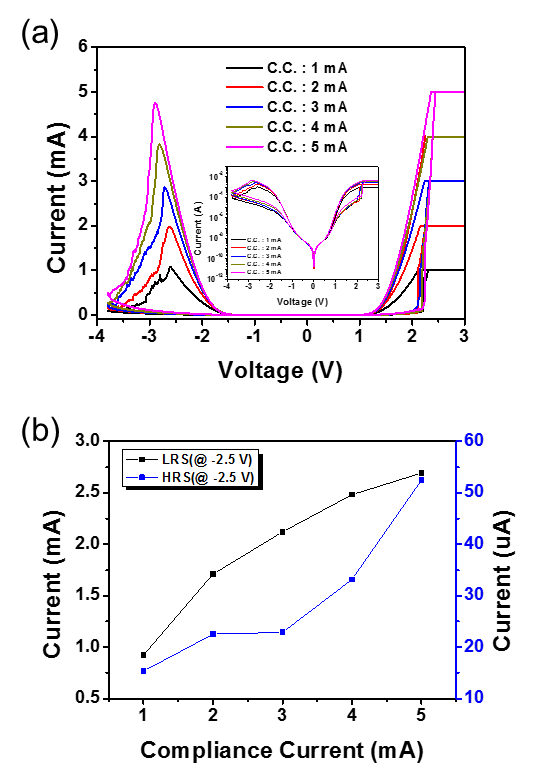
8. Multi-level current operation of 1S1R

Figure S8. (a) Multi-level resistive switching behaviors of 1S1R frames taken at various compliance currents ranging from 1 to − 5 mA. (b) LRS and HRS current values measured at various compliance currents. The inset in (a) plots the semi-log I-V curves ensuring multi-level resistive switching features.


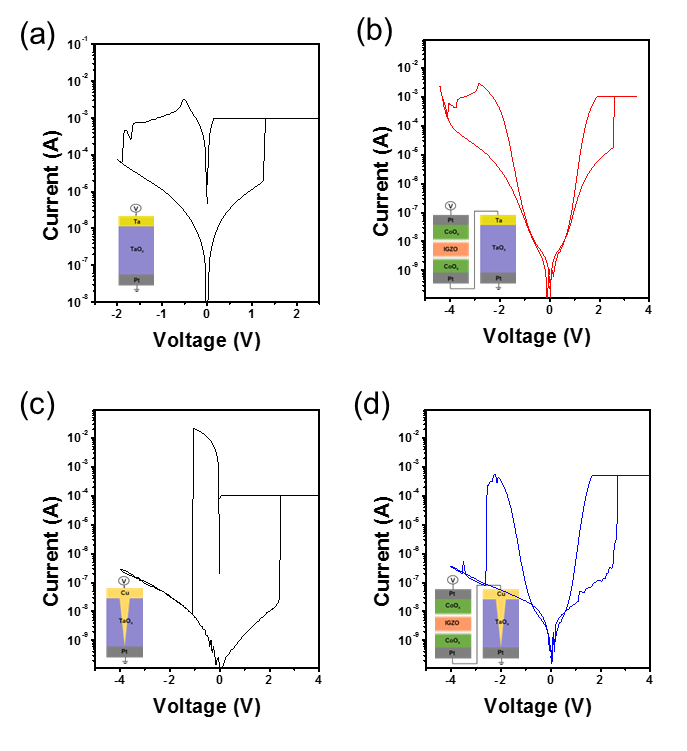
9. Electrical characteristics of 1S1R with various 1R memory devices

Figure S9. Typical I-V characteristics of 1S1R frames integrated with various bipolar resistive switching elements. (a) I-V responses of Ta/TaO_x_/Pt switching element. (b) I-V characteristics of 1S1R integrated with a Ta/TaO_x_/Pt element. (c) I-V responses of the Cu/TaO_x_/Pt atomic resistive switching element. (d) I-V characteristics of 1S1R combined with a Cu/TaO_x_/Pt switching element. The insets in (a) to (d) show the schematics of 1R and 1S1R frames used for measurements.

10. Size dependent I-V features


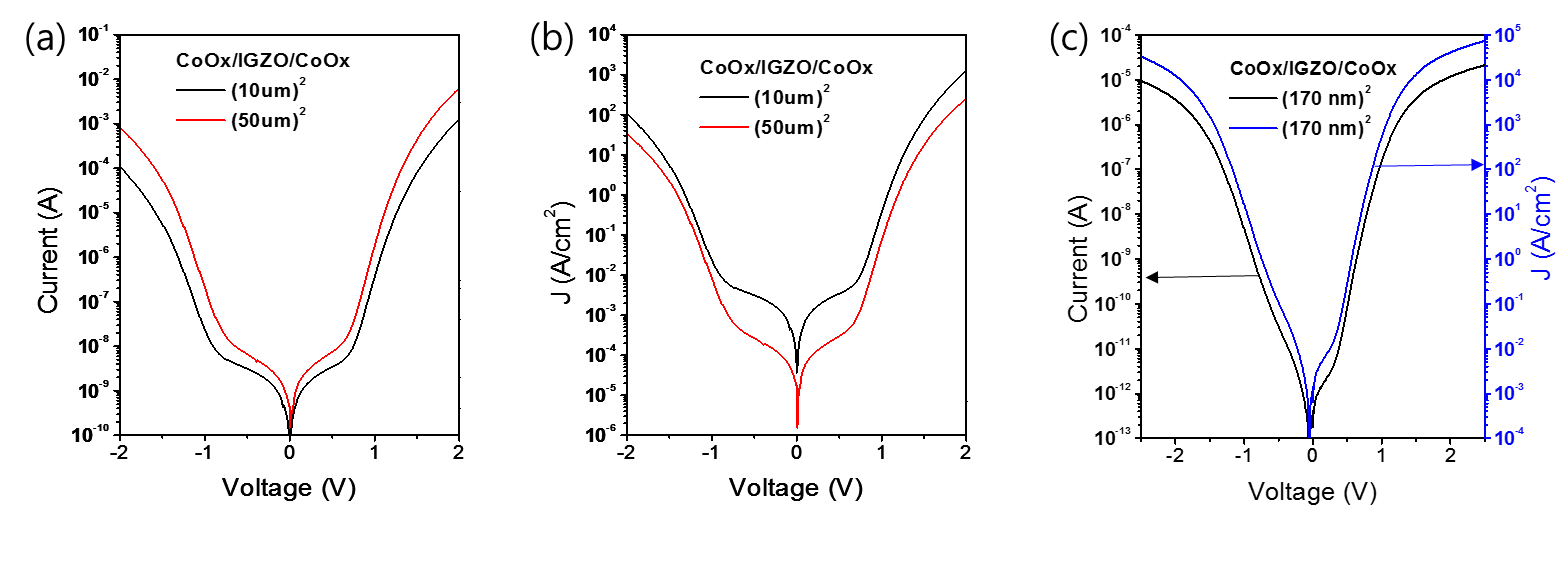


Figure S10. Size dependence of electrical characteristics of pnp selectors. (a) current-voltage and (b) current density-voltage behaviors of 10 um and 50 um device sizes. (c) current-voltage (black line) and current density-voltage (blue line) curves of 170 nm nano-scaled pnp selector.


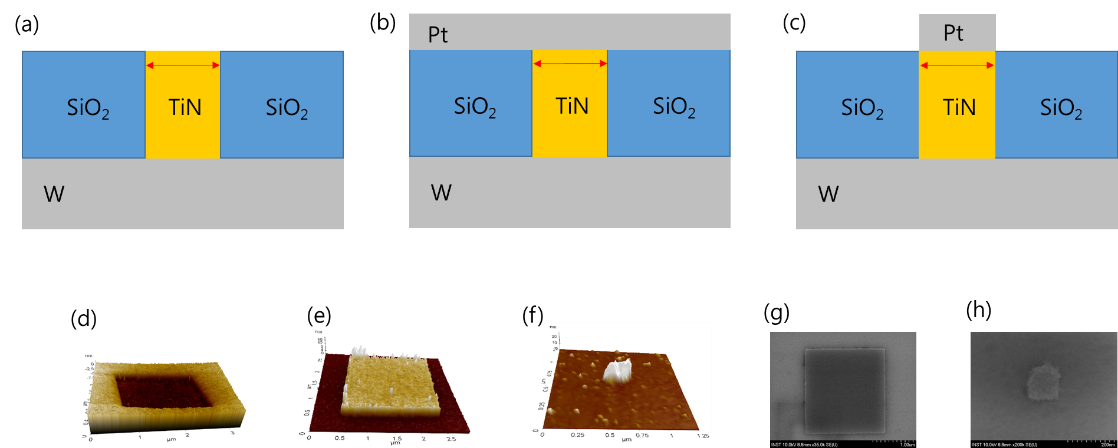


Figure S11. Schematic fabrication flow chart and corresponding AFM/SEM images of 170 nm nano-scaled pnp selectors. (a) Initially prepared 170 nm TiN nano-plug wafer. (b) Deposition of 10 nm-thick Pt layer on TiN wafer. (c) Formation of nano-scaled Pt bottom electrode after ultrasonic treatment. (d-f) Corresponding AFM images of initially TiN nano-plug wafer and patterned Pt electrode. (g) and (h) SEM images of patterned Pt electrodes for 2um and 170 nm-scaled Pt bottom electrode.

To clarify the effect of device size on electrical features of pnp selectors, we have also fabricated the 10×10 um^2^ device by using the same photolithography and lift-off process. As shown in Fig. S11 (a) and (b) below, the current density increased a little with slightly shrinking device size. In addition, to further predict the current density issue in a nano-scaled device, we have also prepared a nano-scaled device by using an unusual method. As mentioned above, due to processing difficulties in direct nano-scaled device fabrication, we have used the nano-plug patterned TiN wafers. The schematic illustrations of nano-plug patterned TiN wafers we have used are given in Figure S11. As seen in this figure, initially nano-scaled TiN plugs (170 nm width in our work) prepared from semiconductor industries as a joint-work were surrounded by insulating SiO_2_ layer. In our process, at first, a 10nm-thick Pt layer was deposited on the TiN plug wafer with the sputtering system as a bottom Pt electrode, as shown in Fig. S11 (b). And then, to form the nano-sized Pt bottom electrode, a proper ultrasonic treatment was conducted in acetone, methanol, and de-ionized (DI) water for several minutes. High work function difference between Pt and SiO_2_ kept the Pt electrode on TiN layer, while the Pt electrode on SiO_2_ part was removed. After the development of nano-scaled Pt bottom electrode, the same fabrication used for micro-sized devices was adapted for the nano-scaled pnp selector.


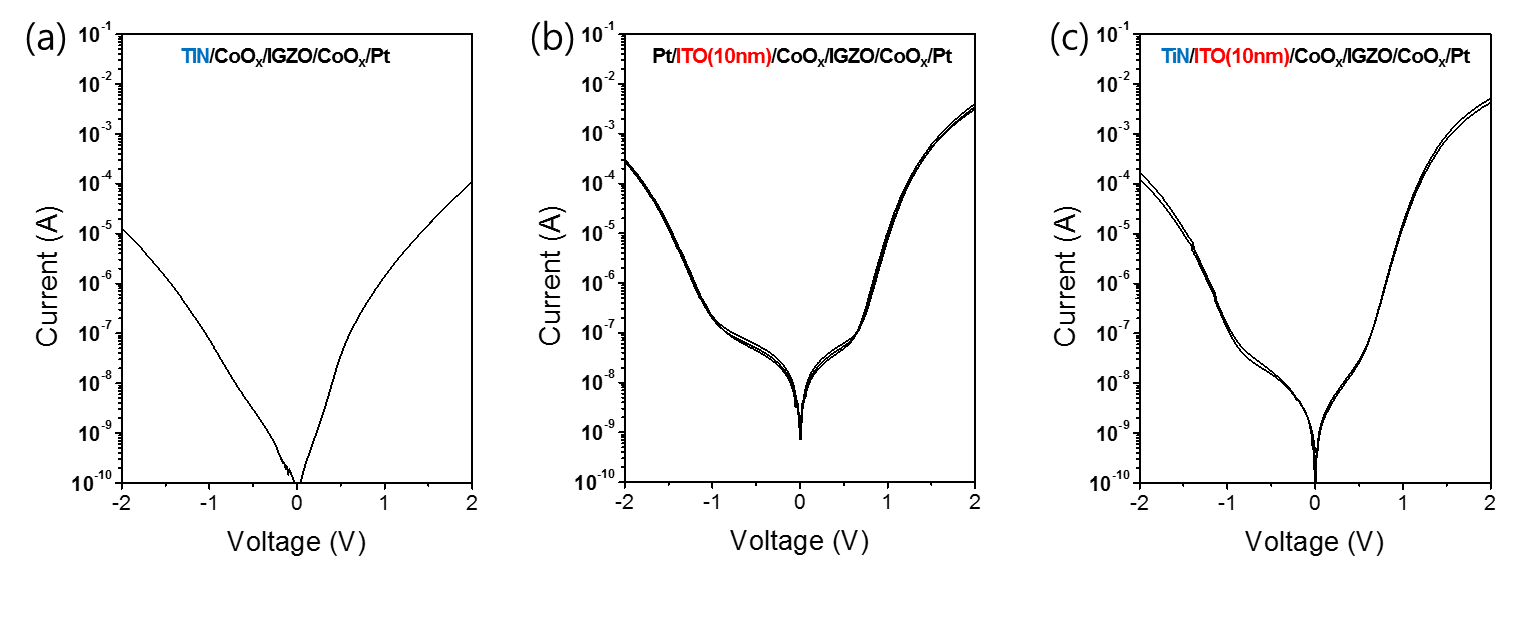
11. Electrode-dependent I-V features of p-n-p selector

Fig. S12. Electrical characteristics of pnp selector with various electrodes. (a) TiN, (b) ITO inserted Pt and (c) TiN electrode.

To clarify the suitability of oxide p-n-p selectors for the complementary metal-oxide semiconductor system, a TiN electrode was also examined. As represented in Fig. S12a, the TiN electrode with a reactive characteristic was highly susceptible to reaction with bottom or Top CoO_x_ layers, resulting in the degraded electrical performance. Thus, the widespread use of TiN electrode may not be appropriate for oxide selector at this moment. To solve this issue, one possible way is the insertion of a highly conductive oxide electrode between TiN electrode and oxide materials to block the possible reaction of oxide materials with a reactive electrode. Fig. S12b and c reveal the compared I-V features of pnp selectors involving Pt and TiN bottom electrode after the insertion of 10 nm-thick tin-doped indium oxide (ITO) layer. As seen in these figure, the I-V characteristics were maintained without significant degradation. Therefore, we anticipate that the use of highly conductive oxide electrode will spur progress towards further development of oxide selectors, even though more works are required in the future.
